# Alcohol-associated liver disease increases the risk of muscle loss and mortality in patients with cirrhosis

**DOI:** 10.1007/s00535-024-02137-4

**Published:** 2024-07-28

**Authors:** Tatsunori Hanai, Kayoko Nishimura, Shinji Unome, Takao Miwa, Yuki Nakahata, Kenji Imai, Atsushi Suetsugu, Koji Takai, Masahito Shimizu

**Affiliations:** 1 Department of Gastroenterology/Internal Medicine, Gifu University Graduate School of Medicine, 1-1 Yanagido, Gifu, 501-1194 Japan; 2 Center for Nutrition Support and Infection Control, Gifu University Hospital, Gifu, Japan

**Keywords:** Alcohol-associated liver disease, Liver cirrhosis, Mortality, Muscle loss rate, Sarcopenia

## Abstract

**Background:**

Rapid skeletal muscle loss adversely affects the clinical outcomes of liver cirrhosis. However, the relationships between the annual changes in skeletal muscle area (ΔSMA/year) and the etiology of cirrhosis, factors associated with muscle loss, and risk of mortality remains unclear.

**Methods:**

A total of 384 patients who underwent multiple computed tomography (CT) scans between March 2004 and June 2021 were enrolled in this study (median age, 67 years; 64% men; median model for end-stage liver disease score, 9). Body composition and ΔSMA/year were estimated using a 3D image analysis system and data from at least two distinct CT scans. Differences in ΔSMA/year among different etiologies of cirrhosis, factors associated with rapid muscle loss (defined as ΔSMA/year ≤  − 3.1%), and the association between ΔSMA/year and mortality were examined.

**Results:**

Patients with alcohol-associated liver disease (ALD) cirrhosis experienced more rapid muscle loss (ΔSMA/year, − 5.7%) than those with hepatitis B (ΔSMA/year, − 2.8%) and hepatitis C cirrhosis (ΔSMA/year, − 3.1%). ALD cirrhosis was independently associated with ΔSMA/year ≤  − 3.1% after adjusting for age, sex, and liver functional reserve. Over a median follow-up period of 3.8 years, ALD cirrhosis, ΔSMA/year ≤  − 3.1%, and low subcutaneous adipose tissue level were found to be significantly associated with reduced survival. ALD cirrhosis (hazard ratio [HR], 2.43; 95% confidence interval [CI] 1.12–5.28) and ΔSMA/year ≤  − 3.1% (HR, 3.68; 95% CI 2.46–5.52) were also predictive of mortality.

**Conclusions:**

These results suggest that ALD cirrhosis increases the risk of rapid muscle loss and mortality in affected patients.

**Supplementary Information:**

The online version contains supplementary material available at 10.1007/s00535-024-02137-4.

## Introduction

Progressive and generalized loss of skeletal muscle mass is a major life-threatening complication of liver cirrhosis (LC) [1]. This phenomenon is highly prevalent among patients with LC, and its incidence in this population is significantly higher than that among patients with other chronic diseases or cancer [2]. Moreover, it increases the risk of adverse outcomes such as falls, fractures, infections, hepatic encephalopathy (HE), and hepatic decompensation and negatively affects clinical outcomes such as treatment efficacy for LC as well as hepatocellular carcinoma (HCC), overall survival, waiting-list and post-liver transplantation mortality, and rates of perioperative complications [3–6]. Recent evidence shows that nutritional interventions, such as branched-chain amino acids (BCAAs) supplementation, exercise therapy, and their combination, may prevent or ameliorate muscle atrophy and improve the prognosis of patients with cirrhosis [7–13]. Therefore, identifying patients at high risk of rapid skeletal muscle loss and susceptibility to sarcopenia is an important step in the efficient implementation of therapeutic interventions for LC.

The time-course change in skeletal muscle area per year (ΔSMA/year) is considered as an important factor for estimating the risk of adverse outcomes in patients with LC, as muscle mass changes significantly during the clinical course of the disease [14–17]. The annual rate of muscle loss in patients with cirrhosis has been shown to be more than twice that among healthy individuals, and this rate is further accelerated in patients with more advanced liver disease, such as Child–Pugh class C cirrhosis [14]. Accumulating evidence shows that older age, male sex, lower body mass index, increased severity of liver disease, and alcohol-associated liver disease (ALD) are associated with sarcopenia in patients with LC [4, 18]. However, most studies on the factors associated with sarcopenia have focused on the condition of patients at the time of diagnosis, and few have examined the factors involved in the ongoing loss of muscle mass that leads to the development of sarcopenia.

In recent years, the proportion cirrhosis due to alcohol consumption has increased [19], possibly due to significant advances in the treatment viral hepatitis; thus, the increasing number of patients with ALD cirrhosis and their poor prognosis has emerged as a major healthcare problem. Notably, unlike other LC etiologies, ALD cirrhosis is associated with severe muscle loss regardless of liver disease severity [16], and sarcopenia is more common in ALD cirrhosis than in non-ALD cirrhosis [4]. However, the effects of the underlying etiology of cirrhosis, and of ALD in particular, on ΔSMA/year and other clinical outcomes are not well understood.

The aim of this study was to investigate the chronological changes in skeletal muscle mass in patients with ALD and viral cirrhosis. We also sought to identify key factors influencing rapid muscle loss and to assess their impact on the prognosis of patients with LC. The findings of this study could provide answers to important clinical questions and facilitate the development of more effective strategies for the treatment of cirrhosis-related muscle loss by providing a basis for identifying high-risk and poor prognosis patient groups.

## Methods

### Study design, ethical considerations, and outcomes

This retrospective cohort study included 384 patients with cirrhosis but without HCC at enrollment who underwent multiple computed tomography (CT) scans at Gifu University Hospital between March 2004 and June 2021. Data on patient clinical characteristics and laboratory results were collected within 1 month of the first CT scan. Patients were followed-up on an outpatient basis every 1 to 3 months at the discretion of their physicians in accordance with the standard clinical practice guidelines for cirrhosis [20, 21] until death or December 31, 2023. Informed consent was obtained from all participants using the opt-out method. The study protocol conformed to the ethical principles of the 1964 Declaration of Helsinki and its subsequent amendments and was reviewed and approved by the Ethics Committee of the Gifu University Graduate School of Medicine (Approval No. 2024-038).

The primary aim of this study was to assess ΔSMA/year in patients with ALD, hepatitis B virus (HBV) cirrhosis, or hepatitis C virus (HCV) cirrhosis. The secondary aims were to identify factors associated with rapid muscle loss (defined as ΔSMA/year ≤ –3.1%) [14] and assess whether accelerated loss of muscle mass has a negative impact on the prognosis of patients with cirrhosis.

### Study population

ALD was diagnosed based on a history of long-term excessive alcohol consumption (more than 50 g/day in women and 60 g/day in men) and no other identifiable causes of liver disease [22–24]. LC was diagnosed based on physical examination, laboratory test results, liver imaging features, and histological findings if available. Overt HE was diagnosed based on the West Haven criteria. The severity of liver dysfunction was determined using the model for end-stage liver disease (MELD) and albumin-bilirubin (ALBI) scores.

The eligibility criteria were age ≥ 20 years and a diagnosis of clinically stable ALD, HBV, or HCV cirrhosis. The exclusion criteria were refusal to provide consent for participation in the study, pregnancy, acute liver failure, acute-on-chronic liver failure, history of organ transplantation, history of transjugular intrahepatic portosystemic shunt procedures, active malignancies (including HCC), neurological, psychiatric, or orthopedic disorders that could lead to muscle wasting, and any life-threatening comorbidities (such as severe infection, cardiac failure, respiratory failure, and renal failure).

### Assessment of body composition

Body composition variables were assessed by quantifying the areas of skeletal muscle, subcutaneous adipose tissue, and visceral adipose tissue using cross-sectional CT images at the level of the third lumbar vertebra (L3), as these are considered to be representative of the corresponding whole body values [25]. A 3D image analysis system (Synapse Vincent; Fujifilm, Tokyo, Japan) that can accurately measure the boundaries of specific tissues in Hounsfield units was used for CT data quantification [19]. The corresponding areas (cm^2^) were then normalized to the square of the height (m^2^) of the patient to obtain the following indices (cm^2^/m^2^): skeletal muscle index (SMI), subcutaneous adipose tissue index (SATI), and visceral adipose tissue index (VATI) [26]. L3 level cross-sectional CT measurements were obtained at both the initial and final follow-up assessments, which were at least 3 months apart (median CT scan interval, 29 months; interquartile range, 6–66 months). ΔSMA/year as well as annual changes in subcutaneous adipose tissue area (ΔSATA/year) and visceral adipose tissue area (ΔVATA/year) were calculated as described previously [14, 27].

### Statistical analysis

Continuous variable data are presented as median and interquartile range values and compared using the Mann–Whitney *U* test. Categorical variable data are presented as numbers and percentages and compared using the chi-square test or Fisher’s exact test. Multiple comparisons were performed using the Kruskal–Wallis test and Steel–Dwass post hoc test. Spearman’s rank correlation coefficient was used to assess the relationship between ΔSMA/year and MELD score. Survival over time was estimated using the Kaplan–Meier method and compared using the log-rank test. Predictors of rapid muscle loss were analyzed using univariate and multivariate logistic regression models, and the results are expressed as odds ratios (ORs) with 95% confidence intervals (CIs).

Optimal SATI cut-off values for predicting mortality were established using receiver operating characteristic curve analysis, and the ability of a model to distinguish between outcome groups was assessed using the area under the receiver operating characteristic curve (AUC). The maximum point of the Youden index “sensitivity + specificity − 1” was selected as the optimal cut-off point. Significant predictors of mortality were determined using univariate and multivariate Cox proportional hazard models, and the results are presented as hazard ratios (HRs) and 95% CIs. A multivariate Cox proportional hazards model was also created based on variables of interest such as cirrhosis etiology and ΔSMA, body composition factors significantly associated with mortality (*P* < 0.05) in univariate analysis with, and known risk factors of mortality in patients with cirrhosis such as age, sex, MELD score, and ALBI score. Specifically, we employed two models due to the use of different indicators to assess hepatic reserve function. Model 1 utilized the MELD score as an indicator of liver dysfunction severity, while Model 2 used the ALBI score instead. All tests were two-sided, and the significance level was set at *P* < 0.05. JMP Pro 17 (SAS Institute Inc., Cary, NC, USA) was used for all statistical analyses.

## Results

### Patient characteristics

A total of 384 patients with cirrhosis were included in this study (244 [64%] male, median age: 67 years, median MELD score: 9, median ALBI score: − 1.85). Overall, 174 (45%) patients had ascites, 28 (7%) had overt HE, and 98 (26%) had diabetes mellitus. LC was attributed to either ALD (32%), HBV (11%), or HCV (57%). Patients with ALD cirrhosis were younger, more likely to be male, and had higher MELD scores, SMI, and VATI but lower SATI than those with viral cirrhosis (Table 1).

**Table 1:** Characteristics of the study population

Characteristics	ALD	HBV	HCV	*P* value*
	(*n* = 124, 32%)	(*n* = 41, 11%)	(*n* = 219, 57%)	
Age, years	60 (53 to 70)	68 (59 to 74)	69 (63 to 76)	< 0.001
Men	108 (87)	21 (51)	115 (53)	< 0.001
Body mass index, kg/m^2^	22.6 (20.4 to 24.7)	22.5 (20.9 to 24.6)	23.0 (21.1 to 25.3)	0.250
Diabetes	38 (31)	13 (32)	47 (22)	0.109
Ascites	66 (53)	21 (51)	87 (40)	0.039
Overt HE	9 (7)	4 (10)	15 (7)	0.806
MELD score	10 (8 to 13)	10 (7 to 12)	8 (7 to 10)	0.001
ALBI score	− 1.81 (− 2.27 to − 1.20)	− 1.82 (− 2.42 to − 1.13)	− 1.88 (− 2.36 to − 1.35)	0.411
Albumin, g/dL	3.1 (2.5 to 3.60)	3.0 (2.5 to 3.8)	3.2 (2.6 to 3.7)	0.357
Creatinine, mg/dL	0.75 (0.64 to 1.02)	0.71 (0.55 to 0.80)	0.73 (0.58 to 0.90)	0.047
Sodium, mEq/L	138 (135 to 140)	139 (135 to 141)	139 (138 to 141)	< 0.001
Total bilirubin, mg/dL	1.1 (0.8 to 1.8)	1.0 (0.7 to 1.6)	1.1 (0.8 to 1.6)	0.569
International normalized ratio	1.13 (1.05 to 1.29)	1.22 (1.00 to 1.41)	1.08 (1.02 to 1.17)	0.002
Ammonia, μg/dL	63 (44 to 95)	59 (42 to 73)	58 (42 to 88)	0.364
SMI, cm^2^	45.4 (38.3 to 51.2)	39.9 (35.3 to 46.5)	44.1 (38.4 to 50.5)	0.046
SATI, cm^2^	25.3 (13.4 to 37.4)	42.8 (20.6 to 56.9)	36.2 (20.3 to 59.4)	< 0.001
VATI, cm^2^	39.8 (26.1 to 58.0)	29.6 (17.4 to 44.0)	30.1 (19.4 to 48.6)	0.005

Data are presented as numbers (percentages) or median (interquartile range) values

*ALBI* Albumin-bilirubin; *ALD* alcohol-associated liver disease; *HBV* hepatitis B virus; *HCV* hepatitis C virus; *HE* hepatic encephalopathy; *MELD* model for end-stage liver disease; *SATA* subcutaneous adipose tissue area; *SATI* subcutaneous adipose tissue index; *SMA* skeletal muscle area; *SMI* skeletal muscle index; *VATA* visceral adipose tissue area; *VATI* visceral adipose tissue index

*Continuous and categorical data were compared using the Kruskal–Wallis test and Chi-square test, respectively

### Time-course changes in SMA

**Fig. 1:**
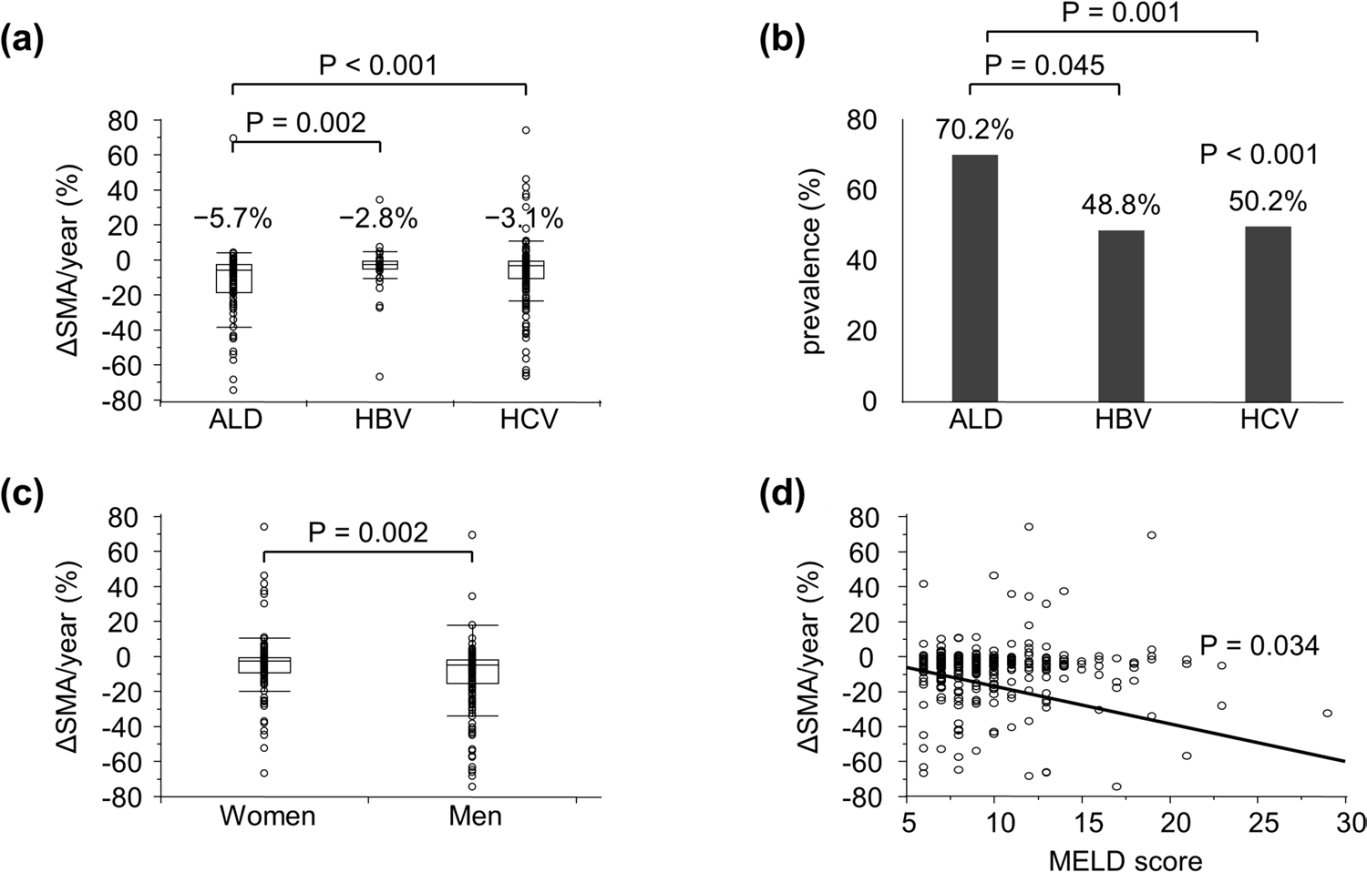
Changes in skeletal muscle area in patients with liver cirrhosis **a** comparison of ΔSMA/year in patients with ALD, HBV, and HCV cirrhosis. **b** Prevalence of ΔSMA/year ≤  − 3.1% for each etiology. **c** Comparison of ΔSMA/year between sexes. **d** Correlation coefficients between ΔSMA/year and MELD score. Data were analyzed using the Mann–Whitney *U* test, Kruskal–Wallis test, Steel–Dwass test, Pearson’s Chi-square test, and Spearman’s rank correlation coefficient. *ALD* alcohol-associated liver disease; *HBV* hepatitis B virus; *HCV* hepatitis C virus; *MELD* model for end-stage liver disease; *ΔSMA* change in skeletal muscle area

ΔSMA/year was − 5.7%, − 2.8%, and − 3.1% for ALD, HBV, and HCV cirrhosis, respectively. These findings suggest that patients with ALD cirrhosis have more rapid skeletal muscle wasting than those with HBV or HCV cirrhosis (Fig. 1a). There were no significant differences between the three groups in terms of ΔSATA/year or ΔVATA/year. Additionally, the prevalence of rapid muscle loss (ΔSMA/year ≤  − 3.1%) was significantly higher among patients with ALD cirrhosis (70.2%) than among those with HBV (48.8%) and HCV (50.2%) cirrhosis (Fig. 1b). Male patients had faster muscle loss (ΔSMA/year, − 4.5%) than female patients (ΔSMA/year, − 2.8%) (Fig. 1c). Furthermore, ΔSMA/year was negatively correlated with the severity of liver disease (*r* =  − 0.11, *P* = 0.034) (Fig. 1d). In addition, we investigated the association between ΔSMA/year and both Child–Pugh classification and ALBI classification. The results showed a negative correlation between ΔSMA/year and both Child–Pugh classification (*P* = 0.004) and ALBI classification (*P* = 0.014).

### Predictors of rapid muscle loss

Seven variables were found to be significantly associated with ΔSMA/year ≤  − 3.1%—ALD, sex, ascites, MELD score, international normalized ratio, SMI, and SATI. Multivariate analysis showed that ALD (OR, 2.25; 95% CI 1.05–4.81 vs. HBV and OR, 2.19; 95% CI 1.30–3.69 vs. HCV), age (OR, 1.03; 95% CI 1.01–1.05), male sex (OR, 1.71; 95% CI 1.08–2.71), and MELD score (OR, 1.08; 95% CI 1.01–1.15) were be significantly associated with ΔSMA/year ≤  − 3.1% in patients with LC (Table 2).

**Table 2:** Multivariate model for rapid muscle loss

	OR (95% CI)	*P* value
Age	1.03 (1.01–1.05)	0.007
Men vs. women	1.71 (1.08–2.71)	0.022
ALD vs. HBV	2.25 (1.05–4.81)	0.036
ALD vs. HCV	2.19 (1.30–3.69)	0.003
MELD score	1.08 (1.01–1.15)	0.017

*ALD* Alcohol-associated liver disease; *CI* confidence interval; *HBV* hepatitis B virus; *HCV* hepatitis C virus; *MELD* model for end-stage liver disease; *OR* odds ratio

### Rapid muscle loss and overall survival

**Fig. 2:**
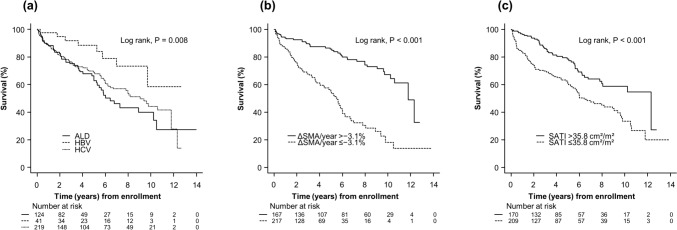
Survival curves for patients with **a** ALD, HBV, and HCV cirrhosis, **b** ΔSMA/year) ≤  − 3.1% and >  − 3.1%, and **c** high (> 35.8 cm^2^/m^2^) and low SATI (≤ 35.8 cm^2^/m^2^). Survival over time was estimated using the Kaplan–Meier method and compared using the log-rank test. *ALD* alcohol-associated liver disease; *HBV* hepatitis B virus; *HCV* hepatitis C virus; *ΔSMA* change in skeletal muscle area; *SATI* subcutaneous adipose tissue index

Patients were followed up for a median of 3.8 years (interquartile range, 1.5–6.8 years) until either death (*n* = 141) or censoring (*n* = 243). None of the patients underwent liver transplantation during the follow-up period. Of the 141 patients who died, 89 died of liver failure, 25 of HCC, 4 of infection, 3 of non-hepatic malignancy, 2 of variceal bleeding, and 18 of other causes. Patients with ALD cirrhosis had a higher risk of mortality than those with HBV cirrhosis (*P* = 0.008, Fig. 2a), with an HR of 2.76 (95% CI 1.31–5.84). Patients with ΔSMA/year ≤  − 3.1% had a higher risk of mortality than those with ΔSMA/year >  − 3.1% (*P* < 0.001, Fig. 2b), with an HR of 3.80 (95% CI 2.60–5.56). The 1-, 3-, 5-, and 10-year survival probabilities among patients with ΔSMA/year ≤  − 3.1% were 86%, 67%, 53%, and 14%, respectively, as compared with 93%, 89%, 85%, and 67%, respectively, among those with ΔSMA/year >  − 3.1%. In a subgroup analysis by etiology, patients with ΔSMA/year ≤  − 3.1% had a lower overall survival rate than those with ΔSMA/year >  − 3.1% for ALD cirrhosis as well as HBV and HCV cirrhosis (Supporting Information Fig. S1).

In the overall cohort, SATI ≤ 35.8 cm^2^/m^2^ was found to be independently associated with mortality (AUC: 0.60, 95% CI 0.54–0.66, *P* < 0.01). Patients with SATI ≤ 35.8 cm^2^/m^2^ had a higher risk of mortality than those with SATI > 35.8 cm^2^/m^2^ (*P* < 0.001, Fig. 2c), with an HR of 2.02 (95% CI 1.42–2.87). The 1-, 3-, 5-, and 10-year survival probabilities were 83%, 69%, 60%, and 33% among patients with SATI ≤ 35.8 cm^2^/m^2^, compared with 96%, 85%, 77%, and 59% among those with SATI > 35.8 cm^2^/m^2^, respectively. In a subgroup analysis, a low level of adipose tissue was associated with increased mortality in both male (*P* = 0.039) and female patients (*P* < 0.001).

### Rapid muscle loss and mortality in patients with LC

ΔSMA/year, ALD, ascites, overt HE, MELD score, ALBI score, international normalized ratio, SATI, and albumin, creatinine, sodium, total bilirubin, and ammonia levels were found to be significant risk factors for mortality according to the univariate Cox regression analysis. As shown in Table 3, the multivariate analysis model 1 identified the following risk factors: ΔSMA/year ≤  − 3.1% (HR, 3.68; 95% CI 2.46–5.52), SATI ≤ 35.8 cm^2^/m^2^ (HR, 1.70; 95% CI 1.17–2.47), and ALD vs. HBV (HR, 2.43; 95% CI 1.12–5.28), independent of the MELD score in patients with LC; multivariate model 2 identified ΔSMA/year ≤  − 3.1% (HR, 3.73; 95% CI 2.48–5.62), SATI ≤ 35.8 cm^2^/m^2^ (HR, 1.55; 95% CI 1.07–2.25), and ALD vs. HBV (HR, 2.86; 95% CI 1.30–6.29) as being significantly associated with mortality independent of the ALBI score.

**Table 3:** Prognostic factors among patients with cirrhosis

Characteristics	HR (95% CI)	*P* value
Model 1		
ALD vs. HBV	2.43 (1.12–5.28)	0.025
ALD vs. HCV	0.76 (0.51–1.12)	0.165
MELD score	1.14 (1.10–1.19)	< 0.001
ΔSMA/year ≤ − 3.1%	3.68 (2.46–5.52)	< 0.001
SATI ≤ 35.8 cm^2^/m^2^	1.70 (1.17–2.47)	0.005
Model 2		
ALD vs. HBV	2.86 (1.30–6.29)	0.009
ALD vs. HCV	0.89 (0.59–1.35)	0.586
ALBI score	2.55 (2.01–3.24)	< 0.001
ΔSMA/year ≤ − 3.1%	3.73 (2.48–5.62)	< 0.001
SATI ≤ 35.8 cm^2^/m^2^	1.55 (1.07–2.25)	0.021

*ALBI* Albumin-bilirubin; *ALD* alcohol-associated liver disease; *CI* confidence interval; *HBV* hepatitis B virus; *HCV* hepatitis C virus; *HR* hazard ratio; *MELD* model for end-stage liver disease; *SATI* subcutaneous adipose tissue index; *ΔSMA* change in skeletal muscle area

We further investigated the synergistic impact of the high-risk group (defined as ΔSMA/year ≤ –3.1% and SATI ≤ 35.8 cm^2^/m^2^) on the prognosis in all subjects and in each etiology. The results showed that mortality in the total cohort was significantly higher in the high-risk group than in the other groups (HR, 3.24; 95% CI 2.23–4.70). Similar results were found in the ALD (HR, 3.16; 95% CI 1.71–5.84) and HCV cirrhosis (HR, 3.47; 95% CI 2.08–5.77) groups, but no such association was found in the HBV cirrhosis group (HR, 1.03; 95% CI 0.16–6.69) (new Supplementary Table 1).

## Discussion

Muscle loss is a significant predictor of mortality in patients with LC and should be actively assessed in routine clinical practice [1]. In particular, it is important to identify patients at high risk of rapid muscle loss leading to sarcopenia [28]. Many studies focused on the incidence of sarcopenia [18], but our study, which comprehensively examined the longitudinal loss of muscle mass and its associated factors, revealed two important findings. The first is that ALD cirrhosis was associated with a two-fold faster decline in skeletal muscle mass than viral cirrhosis. Moreover, ALD cirrhosis, older age, male sex, and advanced liver disease were all independently associated with an increased risk of rapid muscle loss. The second finding was that ALD cirrhosis, rapid muscle loss, and low subcutaneous adipose tissue level were associated with an increased risk of mortality independently of well-established prognostic factors such as MELD and ALBI scores.

The annual rate of muscle loss in younger adults is about 0.5%, compared with 1.0% in older adults [29]. In patients with chronic liver disease, the annual rate of skeletal muscle loss increases with the severity of cirrhosis progression (from − 1.3% in Child–Pugh class A patients to − 3.5% in class B and − 6.1% in class C patients) [14, 16, 27]. Sarcopenia affects 30–70% of patients with LC, with the prevalence varying according to the underlying causes [1, 28]. For example, the prevalence of sarcopenia in cirrhosis due to ALD is 80%, compared with 10–40% in cirrhosis due to non-alcoholic steatohepatitis, viral hepatitis, or autoimmune hepatitis [16, 24]. The present findings, showing that the rate of skeletal muscle loss is significantly faster in ALD cirrhosis (− 5.7%) than in cirrhosis caused by HBV (− 2.8%) or HCV (− 3.1%) and that 70% of patients with ALD cirrhosis were in the poor prognosis group due to skeletal muscle loss, are in agreement with those of previous reports.

The present findings of close associations between rapid muscle loss and ALD cirrhosis, older age, male sex, and advanced liver dysfunction are also consistent with those of previous studies [16, 17]. Meta-analyses have also shown an association between ALD, male sex, and Child–Pugh class C cirrhosis and sarcopenia [4]. Patients with ALD cirrhosis often present with accelerated starvation, malnutrition, and severe liver dysfunction at the time of diagnosis [3, 28], suggesting a propensity for early muscle wasting and a higher susceptibility to sarcopenia. Excessive alcohol consumption may also lead to alcohol-related muscle myopathy [30], which potentially causes rapid muscle loss compared to other etiologies and affects the results of our study. Ethanol and acetaldehyde also increase the levels of inflammatory cytokines and endotoxins, impair hepatic ureagenesis and mitochondrial function, and increase autophagy and levels of muscle ammonia as well as myostatin, a negative regulator of skeletal muscle growth [31, 32]. These factors result in impaired protein synthesis and increased proteolysis, leading to accelerated muscle breakdown and subsequent sarcopenia. In addition, sarcopenia and liver dysfunction may also contribute to muscle loss in cirrhosis [22, 28]. In addition, HCC development and its treatment may affect skeletal muscle mass and prognosis [11, 12]. In this regard, we analyzed the impact of HCC development during the CT scan interval on the ΔSMA/year. Among the total cohort of patients, 85 (22%) developed HCC during this period. The results showed no significant difference in the ΔSMA/year between patients with and without HCC occurrence (*P* = 0.104). We hypothesize that the small number of patients with HCC had a limited impact on the ΔSMA/year.

In the present study, ALD was an independent predictor of mortality in patients with cirrhosis. ALD is the leading cause of cirrhosis, liver failure, and liver-related mortality worldwide [19]. Furthermore, mortality due to ALD could double by 2040 without effective interventions to reduce alcohol consumption [33], making alcohol management an important health issue. Rapid skeletal muscle mass loss (less than -3.1%/year) was also associated with mortality in patients with cirrhosis, which is consistent with a previous report [14]. A prospective cohort study has also suggested that changes in muscle mass may independently predict the development of cirrhosis complications [15]. Furthermore, the coexistence of sarcopenia and rapid muscle loss predicts long-term mortality in patients with cirrhosis, independent of liver function reserve and portal hypertension [14, 17]. Strategies to improve survival in patients with cirrhosis may therefore include treatment to ameliorate progressive muscle loss, improvement of nutritional status and liver functional reserves, and abstinence from alcohol. Clinical trials have shown that nalmefene, an opioid system modulator, reduces the total amount of alcohol consumption by over 60% [34] and improves liver stiffness and hepatic steatosis as measured by transient elastography [35]. Since alcohol reduction contributes to the improvement of liver‐related complications and mortality in patients with ALD cirrhosis, nalmefene may prevent muscle atrophy and improve prognosis [4, 36, 37].

Interventional studies have shown that exercise therapy improves physical ability and increases muscle mass and strength in patients with chronic liver disease, regardless of the presence of HCC [10–13]. A meta‑analysis of randomized controlled trials demonstrates that a combination of aerobic and resistance exercise reduces serious events, such as hepatic failure, HCC, or death, and improves the prognosis of patients with liver cirrhosis [10]. Indeed, a randomized, double-blind trial has shown that a combined nutrition- and exercise-based intervention improved muscle mass in patients with cirrhosis and sarcopenia [38]. As a nutritional therapy, BCAA supplementation has also been shown to improve skeletal muscle mass and liver function reserve and improve the prognosis of patients with cirrhosis [6, 38]. BCAAs not only serve as an energy substrate in patients with liver cirrhosis but also improve liver regeneration, immune function, albumin production, ammonia metabolism, and insulin sensitivity. Accumulating evidence demonstrates that these beneficial physiological effects may prevent hepatocarcinogenesis, inhibit sarcopenia, and improve the prognosis of patients with liver cirrhosis [8, 9]. Given this background, we further analyzed the data to investigate the effects of BCAA supplementation on the ΔSMA/year. Among the enrolled patients, 195 (51%) received BCAA supplementation. The results showed that the ΔSMA was − 4.2% in patients treated with BCAAs and − 3.7% in those without BCAA supplementation, with no statistically significant difference between the two groups (*P* = 0.21). This seemingly contradictory result may be attributed to more severe liver dysfunction (e.g., higher MELD score) in BCAA-treated patients compared with non-BCAA-treated patients (*P* < 0.001), and to unmeasured variables such as alcohol abstinence, dietary intake and habits, and daily physical activity levels [1].

The results of the present study clarified that loss of subcutaneous adipose tissue in addition to that of muscle mass increased the risk of mortality in patients with LC. Similar findings have been confirmed for patients with hematological malignancies and gastrointestinal, renal, and respiratory cancers [39, 40]. Recent studies also suggest that a low SATI can predict increased portal hypertension, liver-related decompensation, and mortality [26, 41]. As subcutaneous adipose tissue serves as the body’s main energy reserve, a low SATI could indicate a significant depletion of energy reserves due to cirrhosis, which could be one of the reasons for worse clinical outcomes [26].

The present study has several limitations. First, it was a single-center, retrospective study, and was thus limited with regard to the availability of accurate relevant information on skeletal muscle loss, such as regarding alcohol abstinence, alcohol-related muscle myopathy, eradication or suppression of the hepatitis virus infection by antiviral therapy, HCC development and its treatment, dietary intake and habits, and daily physical inactivity [1, 12, 42, 43]. Second, the limited etiologically relevant patient backgrounds may have introduced selection bias. Third, as age-related muscle loss is not uniform throughout the body—for example, the annual rate of leg muscle loss in chronic liver disease is faster than that of the trunk muscles [29]—the results of the present study may not accurately reflect the true rate of skeletal muscle loss in LC. Despite these limitations, the detailed quantification of muscle mass and adipose tissue in a large number of patients (*n* = 384), representation of the full spectrum of LC, use of CT, which is the gold standard for body composition assessment in cirrhosis [1, 28], long-term follow-up of the study cohort for more than 4 years, and the appropriate statistical estimation of the risk of muscle loss and mortality are major strengths of this study.

In conclusion, we provide convincing evidence that the rate of skeletal muscle loss in ALD cirrhosis is faster than that in viral cirrhosis. Our data also suggest a strong association between ALD and rapid skeletal muscle loss, both of which predict mortality in patients with LC, regardless of liver disease severity. However, further prospective studies on larger numbers of patients with various underlying etiologies from multiple centers are needed to validate our findings.

## Supplementary Information

Below is the link to the electronic supplementary material.

Supplementary file1 (DOCX 17 KB)

Supplementary file2 (TIF 636 KB)

Supplementary file3 (DOCX 17 KB)

## Abbreviations

ALBI: Albumin-bilirubin
ALD: Alcohol-associated liver disease
AUC: Area under the receiver operating characteristic curve
BCAA: Branched-chain amino acid
BMI: Body mass index
CI: Confidence interval
CT: Computed tomography
HBV: Hepatitis B virus
HCC: Hepatocellular carcinoma
HCV: Hepatitis C virus
HE: Hepatic encephalopathy
HR: Hazard ratio
HU: Hounsfield units
LC: Liver cirrhosis
MELD: Model for end-stage liver disease
OR: Odds ratio
SATA: Subcutaneous adipose tissue area
SATI: Subcutaneous adipose tissue index
SMA: Skeletal muscle area
SMI: Skeletal muscle index
VATA: Visceral adipose tissue area
VATI: Visceral adipose tissue index

## References

[1] Lai JC, Tandon P, Bernal W, et al. Malnutrition, frailty, and sarcopenia in patients with cirrhosis: 2021 practice guidance by the American Association for the Study of Liver Diseases. Hepatology. 2021;74:1611–44. 34233031 10.1002/hep.32049 PMC9134787

[2] Dasarathy S. Consilience in sarcopenia of cirrhosis. J Cachexia Sarcopenia Muscle. 2012;3:225–37. 22648736 10.1007/s13539-012-0069-3 PMC3505573

[3] Sarin SK, Dhingra N, Bansal A, et al. Dietary and nutritional abnormalities in alcoholic liver disease: a comparison with chronic alcoholics without liver disease. Am J Gastroenterol. 1997;92:777–83. 9149184

[4] Tantai X, Liu Y, Yeo YH, et al. Effect of sarcopenia on survival in patients with cirrhosis: a meta-analysis. J Hepatol. 2022;76:588–99. 34785325 10.1016/j.jhep.2021.11.006

[5] Fialla AD, Israelsen M, Hamberg O, et al. Nutritional therapy in cirrhosis or alcoholic hepatitis: a systematic review and meta-analysis. Liver Int. 2015;35:2072–8. 25645300 10.1111/liv.12798

[6] Hanai T, Shiraki M, Nishimura K, et al. Sarcopenia impairs prognosis of patients with liver cirrhosis. Nutrition. 2015;31:193–9. 25441595 10.1016/j.nut.2014.07.005

[7] Kawaguchi T, Izumi N, Charlton MR, et al. Branched-chain amino acids as pharmacological nutrients in chronic liver disease. Hepatology. 2011;54:1063–70. 21563202 10.1002/hep.24412

[8] Kawaguchi T, Shiraishi K, Ito T, et al. Branched-chain amino acids prevent hepatocarcinogenesis and prolong survival of patients with cirrhosis. Clin Gastroenterol Hepatol. 2014;12:1012-8.e1. 24036055 10.1016/j.cgh.2013.08.050

[9] Kitajima Y, Takahashi H, Akiyama T, et al. Supplementation with branched-chain amino acids ameliorates hypoalbuminemia, prevents sarcopenia, and reduces fat accumulation in the skeletal muscles of patients with liver cirrhosis. J Gastroenterol. 2018;53:427–37. 28741271 10.1007/s00535-017-1370-x

[10] Kawaguchi T, Kawaguchi A, Hashida R, et al. Resistance exercise in combination with aerobic exercise reduces the incidence of serious events in patients with liver cirrhosis: a meta-analysis of randomized controlled trials. J Gastroenterol. 2024;59:216–28. 38159112 10.1007/s00535-023-02060-0

[11] Tsuchihashi J, Koya S, Hirota K, et al. Effects of In-hospital exercise on frailty in patients with hepatocellular carcinoma. Cancers (Basel). 2021;13:194. 33430438 10.3390/cancers13020194 PMC7826707

[12] Hashida R, Kawaguchi T, Koya S, et al. Impact of cancer rehabilitation on the prognosis of patients with hepatocellular carcinoma. Oncol Lett. 2020;19:2355–67. 32194735 10.3892/ol.2020.11345 PMC7039060

[13] Koya S, Kawaguchi T, Hashida R, et al. Effects of in-hospital exercise on liver function, physical ability, and muscle mass during treatment of hepatoma in patients with chronic liver disease. Hepatol Res. 2017;47:E22-34. 27062043 10.1111/hepr.12718

[14] Hanai T, Shiraki M, Ohnishi S, et al. Rapid skeletal muscle wasting predicts worse survival in patients with liver cirrhosis. Hepatol Res. 2016;46:743–51. 26579878 10.1111/hepr.12616

[15] Kim TH, Jung YK, Yim HJ, et al. Impacts of muscle mass dynamics on prognosis of outpatients with cirrhosis. Clin Mol Hepatol. 2022;28:876–89. 36117443 10.3350/cmh.2022.0231 PMC9597226

[16] Welch N, Dasarathy J, Runkana A, et al. Continued muscle loss increases mortality in cirrhosis: impact of aetiology of liver disease. Liver Int. 2020;40:1178–88. 31889396 10.1111/liv.14358 PMC7195232

[17] Jeong JY, Lim S, Sohn JH, et al. Presence of sarcopenia and its rate of change are independently associated with long-term mortality in patients with liver cirrhosis. J Korean Med Sci. 2018;33: e299. 30534029 10.3346/jkms.2018.33.e299 PMC6281953

[18] Tuo S, Yeo YH, Chang R, et al. Prevalence of and associated factors for sarcopenia in patients with liver cirrhosis: a systematic review and meta-analysis. Clin Nutr. 2024;43:84–94. 38016243 10.1016/j.clnu.2023.11.008

[19] DiMartini AF, Leggio L, Singal AK. Barriers to the management of alcohol use disorder and alcohol-associated liver disease: strategies to implement integrated care models. Lancet Gastroenterol Hepatol. 2022;7:186–95. 35026172 10.1016/S2468-1253(21)00191-6 PMC12457915

[20] Yoshiji H, Nagoshi S, Akahane T, et al. Evidence-based clinical practice guidelines for liver cirrhosis 2020. J Gastroenterol. 2021;56:593–619. 34231046 10.1007/s00535-021-01788-x PMC8280040

[21] Yoshiji H, Nagoshi S, Akahane T, et al. Evidence-based clinical practice guidelines for liver cirrhosis 2020. Hepatol Res. 2021;51:725–49. 34228859 10.1111/hepr.13678

[22] Thursz M, Gual A, Lackner C, et al. EASL clinical practice guidelines: management of alcohol-related liver disease. J Hepatol. 2018;69:154–81. 29628280 10.1016/j.jhep.2018.03.018

[23] Rinella ME, Lazarus JV, Ratziu V, et al. A multisociety Delphi consensus statement on new fatty liver disease nomenclature. J Hepatol. 2023;79:1542–56. 37364790 10.1016/j.jhep.2023.06.003

[24] Jophlin LL, Singal AK, Bataller R, et al. ACG clinical guideline: alcohol-associated liver disease. Am J Gastroenterol. 2024;119:30–54. 38174913 10.14309/ajg.0000000000002572 PMC11040545

[25] Shen W, Punyanitya M, Wang Z, et al. Total body skeletal muscle and adipose tissue volumes: estimation from a single abdominal cross-sectional image. J Appl Physiol. 1985;2004(97):2333–8. 10.1152/japplphysiol.00744.2004 15310748

[26] Ebadi M, Tandon P, Moctezuma-Velazquez C, et al. Low subcutaneous adiposity associates with higher mortality in female patients with cirrhosis. J Hepatol. 2018;69:608–16. 29709682 10.1016/j.jhep.2018.04.015

[27] Endo K, Kakisaka K, Kuroda H, et al. Annual changes in grip strength and skeletal muscle mass in chronic liver disease: observational study. Sci Rep. 2023;13:1648. 36717617 10.1038/s41598-023-28528-w PMC9887068

[28] Merli M, Berzigotti A, Zelber-Sagi S, et al. EASL clinical practice guidelines on nutrition in chronic liver disease. J Hepatol. 2019;70:172–93. 30144956 10.1016/j.jhep.2018.06.024 PMC6657019

[29] Mitchell WK, Williams J, Atherton P, et al. Sarcopenia, dynapenia, and the impact of advancing age on human skeletal muscle size and strength; a quantitative review. Front Physiol. 2012;3:260. 22934016 10.3389/fphys.2012.00260 PMC3429036

[30] Simon L, Jolley SE, Molina PE. Alcoholic myopathy: pathophysiologic mechanisms and clinical implications. Alcohol Res. 2017;38:207–17. 28988574 10.35946/arcr.v38.2.05 PMC5513686

[31] Kant S, Davuluri G, Alchirazi KA, et al. Ethanol sensitizes skeletal muscle to ammonia-induced molecular perturbations. J Biol Chem. 2019;294:7231–44. 30872403 10.1074/jbc.RA118.005411 PMC6509515

[32] Dasarathy J, McCullough AJ, Dasarathy S. Sarcopenia in alcoholic liver disease: clinical and molecular advances. Alcohol Clin Exp Res. 2017;41:1419–31. 28557005 10.1111/acer.13425 PMC5553706

[33] Julien J, Ayer T, Bethea ED, et al. Projected prevalence and mortality associated with alcohol-related liver disease in the USA, 2019–40: a modelling study. Lancet Public Health. 2020;5:e316–23. 32504584 10.1016/S2468-2667(20)30062-1

[34] Gual A, He Y, Torup L, et al. A randomised, double-blind, placebo-controlled, efficacy study of nalmefene, as-needed use, in patients with alcohol dependence. Eur Neuropsychopharmacol. 2013;23:1432–42. 23562264 10.1016/j.euroneuro.2013.02.006

[35] Mueller S, Luderer M, Zhang D, et al. Open-label study with nalmefene as needed use in alcohol-dependent patients with evidence of elevated liver stiffness and/or hepatic steatosis. Alcohol. 2020;55:63–70. 10.1093/alcalc/agz078 31713583

[36] Llamosas-Falcón L, Probst C, Buckley C, et al. How does alcohol use impact morbidity and mortality of liver cirrhosis? A systematic review and dose-response meta-analysis. Hepatol Int. 2024;18:216–24. 37684424 10.1007/s12072-023-10584-z PMC10920389

[37] Singal AK, Bataller R, Ahn J, et al. ACG clinical guideline: alcoholic liver disease. Am J Gastroenterol. 2018;113:175–94. 29336434 10.1038/ajg.2017.469 PMC6524956

[38] Hernández-Conde M, Llop E, Gómez-Pimpollo L, et al. Adding branched-chain amino acids to an enhanced standard-of-care treatment improves muscle mass of cirrhotic patients with sarcopenia: a placebo-controlled trial. Am J Gastroenterol. 2021;116:2241–9. 34074812 10.14309/ajg.0000000000001301

[39] Ebadi M, Martin L, Ghosh S, et al. Subcutaneous adiposity is an independent predictor of mortality in cancer patients. Br J Cancer. 2017;117:148–55. 28588319 10.1038/bjc.2017.149 PMC5520211

[40] Camus V, Lanic H, Kraut J, et al. Prognostic impact of fat tissue loss and cachexia assessed by computed tomography scan in elderly patients with diffuse large B-cell lymphoma treated with immunochemotherapy. Eur J Haematol. 2014;93:9–18. 24520908 10.1111/ejh.12285

[41] Rodrigues SG, Brabandt B, Stirnimann G, et al. Adipopenia correlates with higher portal pressure in patients with cirrhosis. Liver Int. 2019;39:1672–81. 31207018 10.1111/liv.14175

[42] Georgiou A, Yannakoulia M, Papatheodoridis GV, et al. Assessment of dietary habits and the adequacy of dietary intake of patients with cirrhosis-the KIRRHOS study. Clin Nutr. 2021;40:3992–8. 34139472 10.1016/j.clnu.2021.04.044

[43] Hanai T, Nishimura K, Unome S, et al. A survey questionnaire evaluating physical activity patterns and determinants in patients with chronic liver disease. J Gastroenterol. 2024;59:45–55. 37843553 10.1007/s00535-023-02047-x

