# Supplementary material for: Alcohol-associated liver disease increases the risk of muscle loss and mortality in patients with cirrhosis
Source: J Gastroenterol. 2024 Jul 28;59(10):932–40. doi: 10.1007/s00535-024-02137-4 (PMC11415521; doi:10.1007/s00535-024-02137-4)
Supplement: Supplementary file 1 — Supplementary file1 (DOCX 17 KB) [file 535_2024_2137_MOESM1_ESM.docx]

**Supporting Information**

**Alcohol-associated liver disease increases the risk of muscle loss and mortality in patients with cirrhosis**

**Journal name:** Journal of Gastroenterology

**Short Title:** ALD and muscle loss

Tatsunori Hanai^1^*, Kayoko Nishimura^2^, Shinji Unome^1^, Takao Miwa^1^, Yuki Nakahata^1^, Kenji Imai^1^, Atsushi Suetsugu^1^, Koji Takai^1^, and Masahito Shimizu^1^

^1^Department of Gastroenterology/Internal Medicine, Gifu University Graduate School of Medicine, Gifu, Japan

^2^Center for Nutrition Support and Infection Control, Gifu University Hospital, Gifu, Japan

***Corresponding author:** hanai.tatsunori.p8@f.gifu-u.ac.jp

**Supplementary Figure**

Fig. S1 Survival curves of patients with ΔSMA/year ≤−3.1% and >−3.1% and (a) ALD cirrhosis, (b) HBV cirrhosis, or (c) HCV cirrhosis

Survival over time was estimated using the Kaplan–Meier method and compared using the log-rank test.

ALD, alcohol-associated liver disease; HBV, hepatitis B virus; HCV, hepatitis C virus; ΔSMA, change in skeletal muscle area
