# Supplementary figures and images for: Alcohol-associated liver disease increases the risk of muscle loss and mortality in patients with cirrhosis
Source: J Gastroenterol. 2024 Jul 28;59(10):932–40. doi: 10.1007/s00535-024-02137-4 (PMC11415521; doi:10.1007/s00535-024-02137-4)

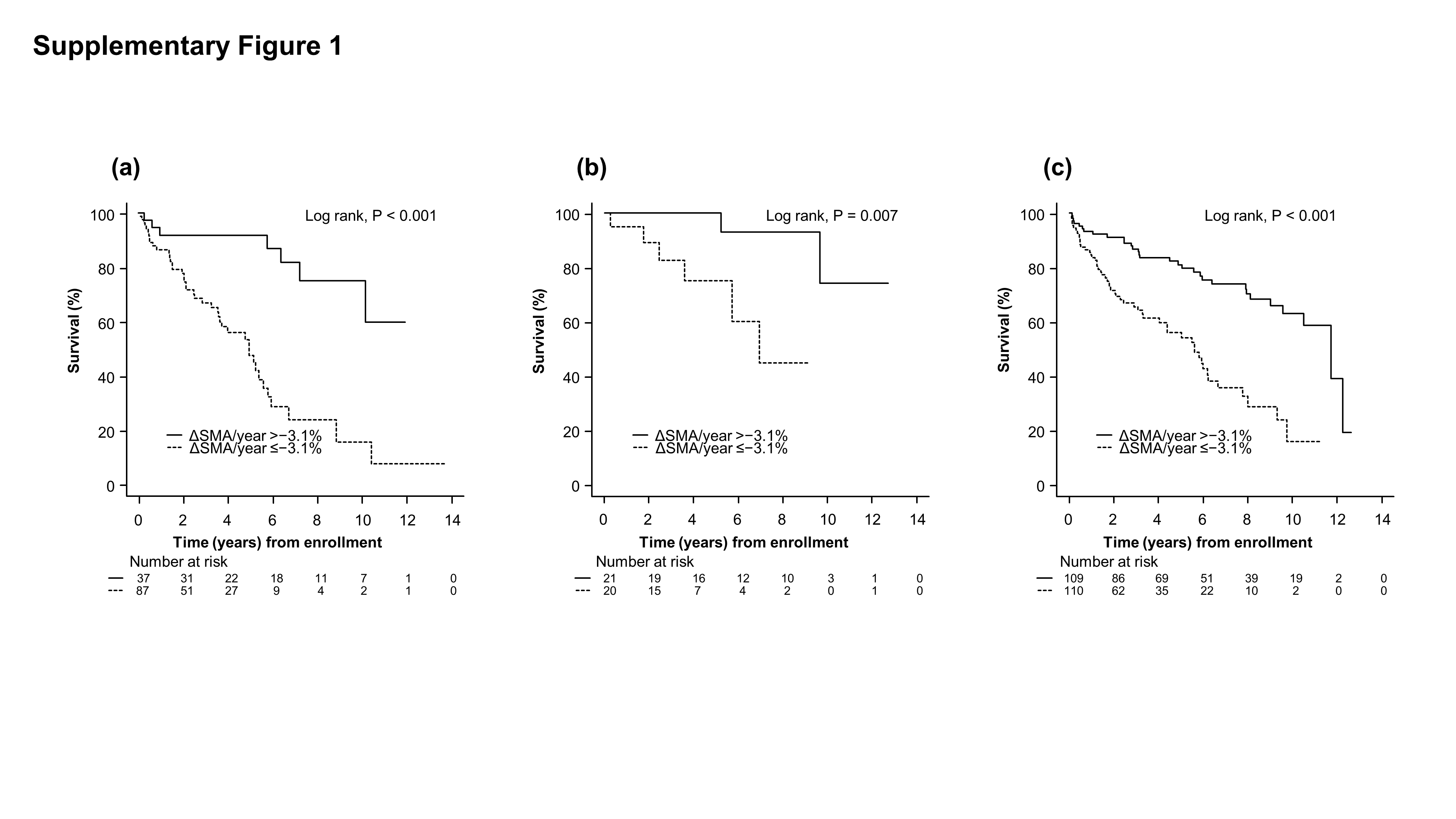

Supplement: Supplementary file 2 — Supplementary file2 (TIF 636 KB) [file 535_2024_2137_MOESM2_ESM.tif]
