# Supplementary material for: Alcohol-associated liver disease increases the risk of muscle loss and mortality in patients with cirrhosis
Source: J Gastroenterol. 2024 Jul 28;59(10):932–40. doi: 10.1007/s00535-024-02137-4 (PMC11415521; doi:10.1007/s00535-024-02137-4)
Supplement: Supplementary file 3 — Supplementary file3 (DOCX 17 KB) [file 535_2024_2137_MOESM3_ESM.docx]

**Supplementary Table 1.** Synergetic impact of rapid muscle loss and low subcutaneous adipose tissue on the prognosis

|  | Total cohort |  | ALD |  | HBV |  | HCV |  |
| --- | --- | --- | --- | --- | --- | --- | --- | --- |
| Characteristics | HR (95% CI) | *P* value | HR (95% CI) | *P* value | HR (95% CI) | *P* value | HR (95% CI) | *P* value |
| ΔSMA/year ≤ –3.1% and SATI ≤35.8 cm²/m²) | 3.24 (2.23–4.70) | <0.001 | 3.16 (1.71–5.84) | <0.001 | 1.03 (0.16–6.69) | 0.978 | 3.47 (2.08–5.77) | <0.001 |
| MELD score | 1.14 (1.10–1.19) | <0.001 | 1.09 (1.02–1.17) | 0.012 | 1.27 (1.01–1.63) | 0.040 | 1.17 (1.11–1.23) | <0.001 |
| ALD vs. HBV | 2.42 (1.11–5.27) | 0.026 |  |  |  |  |  |  |
| ALD vs. HCV | 0.82 (1.79–5.99) | 0.325 |  |  |  |  |  |  |

ALD, alcohol-associated liver disease; CI, confidence interval; HBV, hepatitis B virus; HCV, hepatitis C virus; HR, hazard ratio; MELD, Model for End-Stage Liver Disease; SATI, subcutaneous adipose tissue index; ΔSMA, change in skeletal muscle area
